# Covariate effects and population pharmacokinetic analysis of the anti-FGFR2b antibody bemarituzumab in patients from phase 1 to phase 2 trials

**DOI:** 10.1007/s00280-021-04333-y

**Published:** 2021-08-12

**Authors:** Hong Xiang, Lucy Liu, Yuying Gao, Ago Ahene, Helen Collins

**Affiliations:** 1 Five Prime Therapeutics, Inc., South San Francisco, CA USA; 2 Present Address: Amgen Inc., 1120 Veterans Blvd, South San Francisco, CA 94080 USA; 3 Shanghai Qiangshi Information Technology Co., Ltd., Shanghai, China

**Keywords:** Bemarituzumab, Anti-fibroblast growth factor receptor 2b, Population pharmacokinetics, Gastric and gastroesophageal adenocarcinoma

## Abstract

**Purpose:**

A population pharmacokinetic (PK) analysis of the anti-fibroblast growth factor receptor 2b antibody, bemarituzumab, was performed to evaluate the impact of covariates on the PK and assess whether dose adjustment is necessary for a future phase 3 trial.

**Methods:**

Serum concentration data were obtained from three clinical trials, with 1552 bemarituzumab serum samples from 173 patients, and were analyzed using nonlinear mixed-effects modeling.

**Results:**

A two-compartment model with parallel linear and nonlinear (Michaelis–Menten) elimination from the central compartment best described the bemarituzumab serum concentration data. The final model estimated a typical linear clearance (CL) of 0.311 L/day, volume of distribution in the central compartment (*V*_c_) of 3.58 L, distribution clearance (*Q*) of 0.952 L/day, volume of distribution in the peripheral compartment (*V*_p_) of 2.71 L, maximum drug elimination by nonlinear clearance (*V*_max_) of 2.80 μg/day, and Michaelis–Menten constant (*K*_m_) of 4.45 μg/mL. Baseline body weight, baseline albumin, gender, and chemotherapy were identified as statistically significant covariates on the PK of bemarituzumab. Given the low interindividual variability of bemarituzumab key PK parameters (CL and *V*_c_) and the small or modest effect of all statistically significant covariates on bemarituzumab exposure at steady-state, no covariate is expected to have clinically meaningful effects on bemarituzumab exposure.

**Conclusion:**

No covariate had a clinically meaningful impact on bemarituzumab exposure. These results indicate that dose adjustment of bemarituzumab is not necessary, based on the aforementioned covariates, for a future phase 3 trial in gastric and gastroesophageal junction adenocarcinoma population with FGFR2b overexpression in combination with mFOLFOX6.

**Supplementary Information:**

The online version contains supplementary material available at 10.1007/s00280-021-04333-y.

## Introduction

Bemarituzumab (FPA144) is a first-in-class, recombinant, humanized, afucosylated immunoglobulin G1 (IgG1) kappa monoclonal antibody (mAb) directed against fibroblast growth factor receptor 2 IIIb (FGFR2b) and is being developed by Five Prime Therapeutics as a therapeutic for cancer. Bemarituzumab has two mechanisms of action: blocking the FGFR2b signaling pathway and enhancing antibody-dependent cell-mediated cytotoxicity (ADCC) against FGFR2b-overexpressing tumors.

Bemarituzumab has been evaluated in three clinical trials {FPA144-001, FPA144-002, and FPA144-004 [FIGHT]}. FPA144-001 (NCT02318329), a phase 1, open-label, dose-finding trial evaluating the safety and pharmacokinetics (PK) of FPA144 in patients with advanced solid tumors including gastric and gastroesophageal junction adenocarcinoma (GEA) at six dose levels ranging from 0.3 to 15 mg/kg once every 2 weeks (Q2W), demonstrated evidence of monotherapy activity and acceptable tolerability of bemarituzumab in patients with FGFR2b overexpressing GEA [1]. FPA144-002, a phase 1, open-label, dose-finding trial evaluating the safety and PK of bemarituzumab in Japanese patients with advanced GEA at two dose levels, demonstrated that bemarituzumab can be administered safely to Japanese patients in doses up to 15 mg/kg Q2W. Since GEA tends to be highly heterogeneous within the same tumor, and when present, FGFR2b may not be uniformly distributed throughout the tumor specimen [2, 3], it was projected that combining bemarituzumab with chemotherapeutic agents was likely to improve the clinical benefit over bemarituzumab alone. Therefore, a phase 2, randomized, double-blind, controlled trial evaluating the combination of bemarituzumab with modified FOLFOX6 (mFOLFOX6; leucovorin calcium, fluorouracil, and oxaliplatin) vs placebo with mFOLFOX6 in patients with previously untreated advanced GEA (NCT03343301 and NCT03694522, FIGHT) was conducted. The FIGHT trial using bemarituzumab at 15 mg/kg Q2W with 1 additional dose of 7.5 mg/kg on Cycle 1 Day 8 achieved clinically meaningful and statistically significant improvements across all three of its prespecified efficacy endpoints including objective response rate (ORR), progression-free survival (PFS), and overall survival (OS) in patients with FGFR2b-positive, non-HER2 positive frontline advanced GEA [4].

A population PK (popPK) analysis was originally conducted based on phase 1 FPA144-001 trial alone [5] to support the selection of the dose and schedule for bemarituzumab in the phase 2 FIGHT trial. However, the data available from the phase 1 FPA144-001 trial alone were insufficient for identifying key covariate relationships, such as combination with chemotherapy mFOLFOX6 (combotherapy), tumor type [gastric cancer (GC) vs gastroesophageal junction adenocarcinoma (GEJ)], prior gastrectomy, administration of a single dose of mFOLFOX6 prior to randomization, geographic region, race, and Japanese ethnicity. With the success of the FIGHT trial in patients with GEA, a new popPK analysis using PK data from three trials described above was conducted to evaluate the effects of available covariates on the PK of bemarituzumab in support of a future phase 3 trial in patients with GEA.

The objectives of the current analysis were to develop a popPK model for bemarituzumab to characterize the PK, understand the types of covariates and their magnitude effects on PK parameters, and assess whether dose adjustment for 15 mg/kg Q2W with 1 additional dose of 7.5 mg/kg on Cycle 1 Day 8 is necessary for a future phase 3 trial in combination with mFOLFOX6.

## Materials and methods

### Bemarituzumab serum concentration assay in humans

Bemarituzumab serum concentrations in humans were quantitatively measured at ICON Laboratory Services, Inc. (Whitesboro, NY) with a validated ELISA [5].

### Anti-bemarituzumab antibody assay in humans

The anti-drug antibody levels in study FPA144-001 were determined by a validated bridging electrochemiluminescence assay that utilized Meso Scale Discovery (MSD) technology [5]. The second method was an ELISA with affinity capture and elution pre-purification step to measure samples from studies FPA144-002 and FIGHT. The assay sensitivity was 30.2 ng/mL relative to the rabbit anti-bemarituzumab positive control. Drug tolerance was greater than 200 μg/mL in the presence of 800 ng/mL of rabbit anti-bemarituzumab antibody. Anti-bemarituzumab levels were measured at ICON Laboratory Services, Inc. (Whitesboro, NY).

### Study design and pharmacokinetic population

The popPK analysis was conducted using data from three clinical studies: FPA144-001, FPA144-002, and FIGHT (Supplemental Table 1). All studies were conducted in accordance with the Declaration of Helsinki and Guidelines for Good Clinical Practice; approval from Institutional Review Boards or Independent Ethics Committees was obtained for each trial [6, 7]. Written informed consent was obtained from all trial participants.

Bemarituzumab was administered in a 30 min intravenous (IV) infusion at doses ranging from 0.3 to 15 mg/kg Q2W, with most patients receiving a dose of 15 mg/kg Q2W with or without 1 additional dose of 7.5 mg/kg on Cycle 1 Day 8. Patients were evaluable for PK analysis if they had received at least one dose of bemarituzumab and a corresponding PK sample collection after drug administration. Observations below the lower limit of quantitation (LLOQ) at 0.125 μg/mL were omitted from the analysis.

### Population PK analysis

The popPK analysis was performed using a nonlinear mixed-effects modeling with the first-order conditional estimation with interaction (FOCEI) method [8]. Model parameter estimation and model evaluation were implemented with NONMEM 7, Version 7.4.3 (ICON Development Solutions. Ellicott City, MD, USA) [9] with GNU Fortran 95 Compiler (Version 4.6), Perl-Speaks-NONMEM (PsN) Version 4.2 (Uppsala University, Sweden) [10, 11], and R (version 3.5.3).

### Base model and random-effects model development

Based on known PK properties of bemarituzumab, the default structural model was a two-compartment model with parallel linear and nonlinear (Michaelis–Menten) elimination pathways from the central compartment using the differential equations below [5]:

1 $$\frac{{{\text{dA}}_{c} }}{{{\text{dt}}}} = - \left[ {\left( {\frac{{V_{\max } }}{{K_{m} + \frac{{A_{c} }}{{V_{c} }}}}} \right)/V_{c} + \frac{CL}{{V_{c} }} + \frac{Q}{{V_{c} }}} \right] \times A_{c} + \frac{Q}{{V_{p} }} \times A_{p} $$

2 $$\frac{{{\text{dA}}_{p} }}{{{\text{dt}}}} = \frac{Q}{{V_{c} }} \times A_{c} - \frac{Q}{{V_{p} }} \times A_{p} $$

where *A*_c_ and *A*_p_ are the amounts of drug in central and peripheral compartments, respectively. *V*_max_ represents the maximum drug elimination by nonlinear clearance, and *K*_m_, the Michaelis–Menten constant, indicates the drug concentration at 50% *V*_max_. CL and *Q* represent linear clearance and distribution clearance, respectively, while *V*_c_ and *V*_p_ represent volume of distribution in the central and peripheral compartments, respectively.

Alternative model structures (eg, two-compartment with time-varying clearance) were also explored, as appropriate. No time-varying CL was identified after bemarituzumab administration. The final base model was chosen based on the objective function value (OFV), goodness-of-fit plots, and reliability of model parameter estimates.

Assuming a log-normal distribution, the interindividual variability (IIV) in PK parameters was described by an exponential model:

3 $$\theta_{i} = \exp \left( {\theta_{T} + \eta_{i} } \right)$$

where *θ*_*i*_ is the parameter for the *i*^th^ subject, *θ*_*T*_ is natural logarithm of the typical value of the parameter in the population, and *ƞ*_*i*_ (ETA) is a random interindividual effect with mean 0 and variance *ɷ*^2^. The *ɷ* values are the diagonal elements of the IIV-covariance matrix (Ω), which was initially modeled as diagonal (DIAG option in the NM-TRAN $OMEGA record); thus, assuming no covariance between the random effects. A non-diagonal Ω matrix was finally implemented using the BLOCK option to estimate correlation between CL and *V*_c_ based on the model fitness as well as ETA correlation results.

Residual error was described using an additive error model after log-transformation of the PK data:

4 $$\ln \left( {y_{ij} } \right) = \ln \left( {\hat{y}_{ij} } \right) + \varepsilon_{ij}$$

where *y*_*ij*_ and *ŷ*_*ij*_ represent the *j*th observed and predicted serum concentration, respectively, for the *i*^th^ subject, and *ε* is the random residual effect, which is normally distributed with mean 0 and variance *σ*^2^. Other residual error models were explored if patterns were observed in the individual weighted residual (IWRES) versus individual predicted value (IPRED) plot.

### Covariate model development

Following base model development, covariates likely to impact bemarituzumab PK were explored for a possible correlation with key bemarituzumab post hoc PK parameters (Table 1). These covariates were selected based on physiological plausibility, clinical relevance, availability of data, as well as prior knowledge of similar compounds [5, 12].

**Table 1:** Covariate values of bemarituzumab population pharmacokinetic dataset

Covariate (unit or group)		Studies				All
		FPA144-001 (phase 1)	FPA144-002 (phase 1)	FPA144-004 (phase 1)	FPA144-004 (phase 2)	
No. of patients		79	6	12	76	173
No. of PK samples		906	110	175	361	1552
Continuous covariates {median [minimum, maximum]}						
Age (years)		59 [25, 86]	66 [41, 74]	69 [33, 79]	60 [23, 80]	60 [23, 86]
Weight (kg)		62.3 [35.5, 148]	63.3 [55.9, 73.1]	71.6 [61.2, 89.0]	61.8 [43.5, 118]	63.9 [35.5, 148]
Alkaline phosphatase (U/L)		108 [47.0, 787]	207 [166, 487]	124 [67.0, 338]	95.0 [32.0, 1255]	103 [32.0, 1255]
Albumin (g/L)		38.0 [19.0, 46.0]	36.5 [30.0, 42.0]	38.0 [30.0, 46.0]	39.0 [21.0, 50.2]	38.0 [19.0, 50.2]
Total protein (g/L)		69.0 [47.0, 93.0]	62.0 [59.0, 69.0]	71.0 [64.0, 86.0]	69.0 [43.0, 87.0]	69.0 [43.0, 93.0]
Aspartate transaminase (U/L)		24.0 [7.00, 106]	23.0 [18.0, 33.0]	20.0 [9.00, 102]	21.0 [9.00, 113]	22.0 [7.00, 113]
Alanine aminotransferase (U/L)		18.0 [6.00, 74.0]	14.5 [11.0, 28.0]	15.5 [9.00, 33.0]	17.5 [5.00, 128]	17.0 [5.00, 128]
Total bilirubin (µmol/L)		6.84 [1.71, 34.2]	10.3 [8.55, 13.7]	6.84 [3.25, 22.2]	8.12 [2.57, 25.7]	8.04 [1.71, 34.2]
Estimated glomerular filtration rate (eGFR, mL/min/1.73m^2^)		89.1 [29.1, 145]	92.6 [82.8, 113]	101 [65.4, 126]	98.4 [54.6, 141]	95.1 [29.1, 145]
Lactate dehydrogenase (U/L)		225 [100, 1041]	189 [180, 305]	214 [143, 499]	215 [91.0, 963]	215 [91.0, 1041]
Tumor size (mm)		45.0 [10.0, 210]	–	–	52.0 [11.0, 297]	50.4 [10.0, 297]
Categorical covariates {no. of patients (%)}						
Gender	Male	46 (58.2%)	6 (100%)	10 (83.3%)	51 (67.1%)	113 (65.3%)
	Female	33 (41.8%)	–	2 (16.7%)	25 (32.9%)	60 (34.7%)
Race	White	31 (39.2%)	–	9 (75.0%)	30 (39.5%)	70 (40.5%)
	Asian	46 (58.2%)	6 (100%)	1 (8.3%)	44 (57.9%)	97 (56.1%)
	Other	2 (2.53%)	–	2 (16.7%)	2 (2.63%)	6 (3.47%)
Eastern cooperative oncology group (ECOG)	0	24 (30.4%)	2 (33.3%)	6 (50.0%)	24 (31.6%)	56 (32.4%)
	≥1	55 (69.6%)	4 (66.7%)	6 (50.0%)	52 (68.4%)	117 (67.6%)
Hepatic function based on NCI-ODWG	Normal	52 (65.8%)	5 (83.3%)	10 (83.3%)	63 (82.9%)	130 (75.1%)
	Mild	14 (17.7%)	1 (16.7%)	2 (16.7%)	12 (15.8%)	29 (16.8%)
	Missing	13 (16.5%)	–	–	1 (1.32%)	14 (8.09%)
Renal function	Normal	28 (35.4%)	4 (66.7%)	8 (66.7%)	54 (71.1%)	94 (54.3%)
	Mild	35 (44.3%)	2 (33.3%)	4 (33.3%)	21 (27.6%)	62 (35.8%)
	Moderate	15 (19.0%)	–	–	1 (1.3%)	16 (9.2%)
	Severe	1 (1.3%)	–	–	–	1 (0.6%)
Prior gastrectomy	No	53 (67.1%)	–	12 (100%)	55 (72.4%)	120 (69.4%)
	Yes	26 (32.9%)	–	–	21 (27.6%)	47 (27.2%)
	Missing	–	6 (100%)	–	–	6 (3.47%)
FGFR2b status	2 + /3 + < 10%	–	–	–	31 (40.8%)	31 (17.9%)
	2 + /3 + ≥ 10%	–	–	–	44 (57.9%)	44 (25.4%)
	Missing	79 (100%)	6 (100%)	12 (100%)	1 (1.32%)	98 (56.6%)
Tumor type	GC	48 (60.8%)	6 (100%)	–	65 (85.5%)	119 (68.8%)
	GEJ	5 (6.33%)	–	–	11 (14.5%)	16 (9.25%)
	Other	26 (32.9%)	–	12 (100%)	–	38 (22.0%)
Therapy	Monotherapy	79 (100%)	6 (100%)	–	–	85 (49.1%)
	Combotherapy	–	–	12 (100%)	76 (100%)	88 (50.9%)
mFOLFOX6 prior to randomization	No	79 (100%)	6 (100%)	12 (100%)	41 (53.9%)	138 (79.8%)
	Yes	–	–	–	35 (46.1%)	35 (20.2%)
Geographic region	US/Europe/Australia	34 (43.0%)	–	12 (100%)	32 (42.1%)	78 (45.1%)
	China	–	–	–	14 (18.4%)	14 (8.09%)
	Rest of Asia	45 (57.0%)	6 (100%)	–	30 (39.5%)	81 (46.8%)

*FGFR2b* fibroblast growth factor receptor 2

Given the previously known effect of body weight on the clearance and volume of other antibodies and bemarituzumab [5, 12], the effect of body weight on PK parameters was tested first as part of the base model development. Significant body weight effects were incorporated into the base model based on improvements in model fit. Once the base model was developed, covariate screening was conducted by examining correlations between all other covariates and relevant PK parameters graphically, followed by linear regression (for continuous covariates) and analysis of variance (ANOVA) testing (for categorical covariates) using R. These analyses were conducted using the individual empirical Bayesian estimates (EBEs) of interindividual random effects of PK parameters (ETA values) obtained from the base model. Only those covariates that showed a significant (*p* < 0.05) correlation with the relevant PK parameters that could be meaningfully explained from both a clinical and scientific perspective were examined further in covariate modelling using NONMEM.

Continuous covariates were modeled using a power model as described in the following equation:

5 $$\theta_{i} = \exp \left( {\theta_{T} + k_{{\text{cov}}} \cdot \ln \left( {\frac{{{\text{Cov}}_{i} }}{{{\text{Cov}}_{{{\text{pop}}}} }}} \right) + \eta_{i} } \right)$$

Categorical covariates were modeled using the general equation:

6 $$\theta_{i} = \exp \left( {\theta_{T} + \mathop \sum \limits_{1}^{j} \left( {k_{{\text{cov}}} \cdot \left( {\begin{array}{*{20}c} {0, {\text{if}}\, X_{i} \ne j} \\ {1, {\text{if}}\, X_{i} = j} \\ \end{array} } \right)} \right) + \eta_{i} } \right)$$

where *θ*_*i*_ is the individual parameter value for the *i*th subject, *θ*_*T*_ is the natural logarithm of the typical value of the parameter in the population, and *ƞ*_*i*_ is an interindividual random effect with mean of zero and variance *ɷ*^2^. For a continuous covariate, Cov_*i*_ is the individual covariate value for the *i*^th^ subject, Cov_pop_ is the population median or reference value of the covariate, and *k*_cov_ is the coefficient describing the strength of the covariate effect on the parameter. For a categorical covariate, *X*_*i*_ is the individual categorical covariate indicator for the *i*^th^ subject (whose possible values are indicated by *j*), and *k*_cov*,j*_ is the coefficient describing the strength of the covariate effect for category *j* (which is zero for the reference category).

Selection of the final covariate model (final popPK model) was determined for its significance based on the likelihood ratio test at the *p* < 0.01 level for forward inclusion and *p* < 0.001 for backward deletion.

### Model evaluation

The final popPK model was evaluated with multiple model qualification/validation methods, including goodness-of-fit diagnostics, prediction-corrected visual predictive check (pcVPC) [13], numerical predictive check (NPC) [14], bootstrap [15, 16], and shrinkage assessments [17].

pcVPC was used to visually assess the ability of the model to reproduce both the central tendency and variability of observed data over time [13]. A total of 1000 replicates of the original popPK dataset were simulated using observed covariates and dose regimens for each subject, the final model parameter estimates, subject-specific random effects, and residual error. The simulation-based 95% confidence interval (CI) (calculated as the 2.5th–97.5th percentile of the 1000 simulated trials) of the predicted median, 2.5th, and 97.5th percentiles of the concentration–time profiles and the corresponding observed data, normalized based on the median population prediction of each time bin, were overlaid to assess whether the model predictions can capture the observed median and spread of the concentration–time profiles.

### Sensitivity analyses

The sensitivity analysis was performed for the final popPK model to examine the influence of statistically significant covariates on the predicted exposures including area under concentration–time curve at steady-state (AUC_ss_), maximum serum concentration at steady-state (C_max,ss_), and trough concentration at steady-state (C_troug,ss_) after the target dose of 15 mg/kg Q2W for 52 weeks with 1 additional dose of 7.5 mg/kg bemarituzumab on Cycle 1 Day 8. Tornado plots were generated for different scenarios (10th and 90th values of continuous covariates or possible group of categorical covariates) to show the influence of each covariate on expected exposure compared with the reference value (the predicted exposure in a typical male patient on monotherapy with a body weight of 64 kg and albumin of 38 g/L after the target dose).

### Population PK model simulations

To determine the predicted effect of covariates on steady-state exposures in the GEA population, the bemarituzumab concentration–time profiles were simulated using the EBEs of individual PK parameters based on the final popPK model for 135 GEA patients following the target dose. The predicted steady-state exposure metrics (AUC_ss_, C_max,ss_, and C_troug,ss_) were compared among covariate subgroups to evaluate the need for dose adjustment in patient subgroups of interest, including age group (< 65 vs≥ 65 years), weight quartiles, albumin quartiles, gender, race (White vs Asian vs other), Japanese ethnicity (Japanese vs non-Japanese), geographic region (US, Europe, and Australia vs mainland China vs rest of Asia), FGFR2b status [immunohistochemistry (IHC)-detected FGFR2b ≥ 10% vs FGFR2b 1–9%], therapy [monotherapy (FPA144-001 and FPA144-002) vs combotherapy (FIGHT)], tumor type (GC vs GEJ), renal function categories (estimated glomerular filtration rate [eGFR] ≥ 90, 60–89, 30–59, and 15–29 mL/min), hepatic function classified by National Cancer Institute Organ Dysfunction Working Group (NCI-ODWG) criteria (normal vs mild), Eastern Cooperative Oncology Group (ECOG) (0 vs ≥ 1), prior gastrectomy (yes/no), and administration of a single dose of mFOLFOX6 prior to randomization (yes/no).

## Results

### Population PK analysis dataset

The PK analysis dataset included 1552 bemarituzumab serum concentration–time data points from 173 patients (Table 1). Of these patients, 85 (49.1%) received monotherapy and 88 (50.9%) received combotherapy. Observations below the LLOQ, which made up 1.22% (19/1552) of data points, were omitted in the analysis. No anti-bemarituzumab antibody was detected in any patients post-bemarituzumab treatment in all 3 studies.

### Base model development and covariate assessment

Based on prior popPK analyses of bemarituzumab [5], a two-compartment model with parallel linear and nonlinear (Michaelis–Menten) elimination from the central compartment was chosen as the starting structural model. After the base model structure was established, the model was rerun after excluding outlier data points with absolute conditional weighted residuals (|CWRES|) > 5. Subsequently, given the previously known effect of body weight on the clearance and volumes of other antibodies and bemarituzumab [5, 12], the effects of baseline body weight on CL, *V*_c_, *Q*, and *V*_p_ were examined. The results indicated that adding body weight effect on all four parameters significantly improved the model fit (*p* < 0.01, OFV decreased by 72.2, from − 3200.8 to − 3273.0). However, removing body weight effect from *Q* and *V*_p_ resulted in a nonsignificant change in OFV (*p* = 0.038, increases by 6.558, from − 3273.0 to − 3266.4 for 2 degrees of freedom). Compared to the base model without body weight effects, the model with the body weight effects added to CL and *V*_c_ was established as the final base PK model for bemarituzumab and reduced the IIV of CL and *V*_c_ by 19.7% and 32.9%, respectively.

Based on an examination of PK parameter–covariate relationships, gender, combotherapy/study, aspartate aminotransferase, albumin, a single dose of mFOLFOX6 prior to randomization, hepatic function based on NCI-ODWG, ECOG performance status, lactate dehydrogenase, total bilirubin on CL, gender, and combotherapy/study on *V*_c_ had a statistical significance of *p* < 0.05 and were thus carried forward to the forward covariate search in NONMEM. In addition, tumor type (GC/GEJ/other) was also carried forward to the forward covariate search on both CL and *V*_c_ for further evaluation. Screening of other covariates showed that age, race, eGFR based on Chronic Kidney Disease Epidemiology Collaboration (CKD-EPI) equation [18], alkaline phosphatase, alanine aminotransferase, total protein, tumor size, prior gastrectomy, tumor type, FGFR2b status, and geographic region were not statistically significant covariates on bemarituzumab CL and *V*_c_.

### Final population PK model

The covariate modelling process began with univariate testing, where each covariate effect that was found to be significant in the covariate screening was added to the base model one at a time. Testing of the covariates one at a time using a stepwise forward addition method in NONMEM showed that the effect of albumin and combotherapy/study on CL, and gender on *V*_c_, were significant (*p* < 0.01). The full popPK model included all significant covariate relationships. Covariates were then excluded from the full popPK model one at a time using a stepwise backward elimination method. The criterion for retention was a change in likelihood ratio > 10.83 for one parameter (*p* < 0.001). No covariate was removed in the backward elimination process.

The parameter–covariate relations in the final popPK model are described by the following equations:

7 $$\begin{gathered} {\text{CL}}_{i} \left( {\text{L/hr}} \right) = \exp\, ( - 4.35 + 0.695 \times \ln \left( {\frac{{{\text{WT}}_{i} }}{64}} \right) - 0.657 \times \ln \left( {\frac{{{\text{ALB}}_{i} }}{38}} \right) - \hfill \\ 0.200 \times \left( {{\text{Therapy}}_{i} = {\text{combotherapy}}/{\text{study}}} \right) + \eta_{{{\text{CL}},\,i}}  \hfill \\ \end{gathered}$$

8 $${\text{Vc}}_{i} \left( {\text{L}} \right) = \exp (1.28 + 0.369 \times \ln \left( {\frac{{{\text{WT}}_{i} }}{64}} \right) - 0.164 \times \left( {{\text{Gender}}_{i} = {\text{female}}} \right) + \eta_{Vc,i} $$

where CL_*i*_ and *V*_ci_ represent the linear clearance from the central compartment and volume of distribution of the central compartment of the ith individual; *ƞ*_CL,i_ and *ƞ*_Vc,i_ are the interindividual random effects of CL and *V*_c_ of the ith individual; WT_i_, ALB_i_, Gender_i_, and Therapy_i_ represent the body weight, albumin, gender, and combotherapy/study of the i^th^ individual, respectively.

Parameter estimates for the final popPK model for bemarituzumab are presented in Table 2. The typical values of CL, *V*_c_, *V*_p_, *Q*, *V*_max_, and *K*_m_ were precisely estimated, as evidenced by the small relative standard error (RSE) values (< 13%) and narrow confidence intervals from bootstrapping. The estimated coefficients of body weight, albumin, gender, and combotherapy/study effects were generally estimated with adequate precision (%RSE ranging from 6.46 to 23.2%). The IIVs and residual error were also well estimated, with %RSE ranging from 6.5 to 10.7% for IIVs and 5.19% for residual error.

**Table 2:** Summary of final population pharmacokinetic parameters

Parameter	Parameter description	Base model estimates (% RSE)	Final model estimate (% RSE)	Bootstrap estimates median (95% CI)	Final model shrinkage (%)
exp (*θ*_1_*24	Maximum drug elimination by nonlinear clearance, *V*_max_ (µg/day)	4.03 (17.0%)	2.80 (4.13%)	3.29 (0.77, 10.4)	–
exp (*θ*_2_)	Michaelis–Menten constant, *K*_m_ (µg/mL)	5.63 (15.2%)	4.45 (8.32%)	5.58 (0.665, 39.1)	–
exp (*θ*_3_)*24	Linear clearance, CL (L/day)	0.275 (3.53%)	0.311 (3.68%)	0.306 (0.261, 0.348)	–
*θ*_7_	Influence of body weight on CL	0.703 (4.00%)	0.695 (6.46%)	0.718 (0.518, 0.987)	–
*θ*_9_	Influence of albumin on CL	–	− 0.657 (15.2%)	− 0.659 (− 1.03, − 0.312)	–
*θ*_10_	Influence of combotherapy/study on CL	–	− 0.200 (23.2%)	− 0.205 (− 0.303, − 0.0849)	–
exp (*θ*_4_)	Volume of central compartment, *V*_c_ (L)	3.38 (1.42%)	3.58 (1.64%)	3.58 (3.46, 3.69)	–
*θ*_8_	Influence of body weight on *V*_c_	0.499 (9.92%)	0.369 (13.7%)	0.373 (0.242, 0.491)	–
*θ*_11_	Influence of gender on *V*_c_	–	− 0.164 (15.9%)	− 0.165 (− 0.22, − 0.113)	–
exp (*θ*_5_)*24	Inter-compartmental clearance, *Q* (L/day)	0.953 (13.8%)	0.952 (12.5%)	0.928 (0.684, 1.98)	–
exp (*θ*_6_)	Volume of peripheral compartment, *V*_p_ (L)	2.78 (5.89%)	2.71 (4.92%)	2.71 (2.41, 3.09)	–
*ɷ*^2^Cl, *V*_c_	Covariance between CL and *V*_c_	0.0172 (34.1%)	0.0128 (39.8%)	0.0122 (0.00216, 0.0229)	–
Inter-individual variability (% RSE)*	*V*_max_	89.9 (10.8%)	97.4 (6.84%)	89.8 (58.4, 144)	44.6%
	CL	33.3 (7.24%)	29.2 (6.50%)	27.8 (21.0, 32.8)	13.7%
	*V*_c_	16.4 (6.57%)	14.9 (7.30%)	14.6 (12.2, 16.8)	15.7%
	*V*_p_	59.5 (10.4%)	60.4 (10.7%)	59.3 (45.9, 72.7)	20.5%
*δ*	Residual variability (%)	14.5 (4.95%)	14.6 (5.19%)	14.5 (13.1, 16.2)	14.3%

*CI* confidence interval, *RSE* relative standard error

*IIV is expressed as CV%

For a typical male patient on monotherapy with a body weight of 64 kg and albumin of 38 g/L, the estimated CL was 0.311 L/day, *V*_c_ was 3.58 L, *Q* was 0.952 L/day, *V*_p_ was 2.71 L, *V*_max_ was 2.80 μg/day, and *K*_m_ was 4.45 μg/mL. The estimated linear clearance half-life was 14.9 days. IIVs on CL, *V*_c_, and *V*_p_ were 29.2%, 14.9%, and 60.4%, respectively. The *η*-shrinkage for CL and *V*_c_ was low (13.7% and 15.7%, respectively), suggesting their EBEs could be used to accurately describe the relationships between CL or *V*_c_ and the relevant covariates. The larger IIV (97.4%) and relatively higher η-shrinkage (44.6%) for *V*_max_ were expected given most patients were treated in the linear dose range. The *ε*-shrinkage for the residual error (*ε*) was 14.3%. Based on the estimated coefficients of covariate effects in the final model, a 10% decrease in body weight resulted in 7.06% decrease in CL and 3.81% decrease in *V*_c_ (e.g., a 10% decrease from 64 kg resulted in CL of 0.289 L/day and *V*_c_ of 3.45 L). A 10% decrease in albumin resulted in 7.17% increase in CL (e.g., 10% decrease from 38 g/L resulted in CL of 0.333 L/day). Females (*N* = 60) exhibited 15.1% smaller *V*_c_ compared to males (*N* = 113). Patients on combotherapy (FIGHT, *N* = 88) exhibited 18.1% lower CL than patients on monotherapy (FPA144-001 and FPA144-002, *N* = 85). The inclusion of albumin and combotherapy/study on CL, and gender on *V*_c_ in the final popPK model reduced the IIV of CL by 23.1% and *V*_c_ by 18.4% compared with the base model (*ɷ*^2^_CL_ and *ɷ*^2^_Vc_ are 0.0854 and 0.0221 for the final model vs 0.111 and 0.0270 for the base model, respectively).

**Fig. 1:**
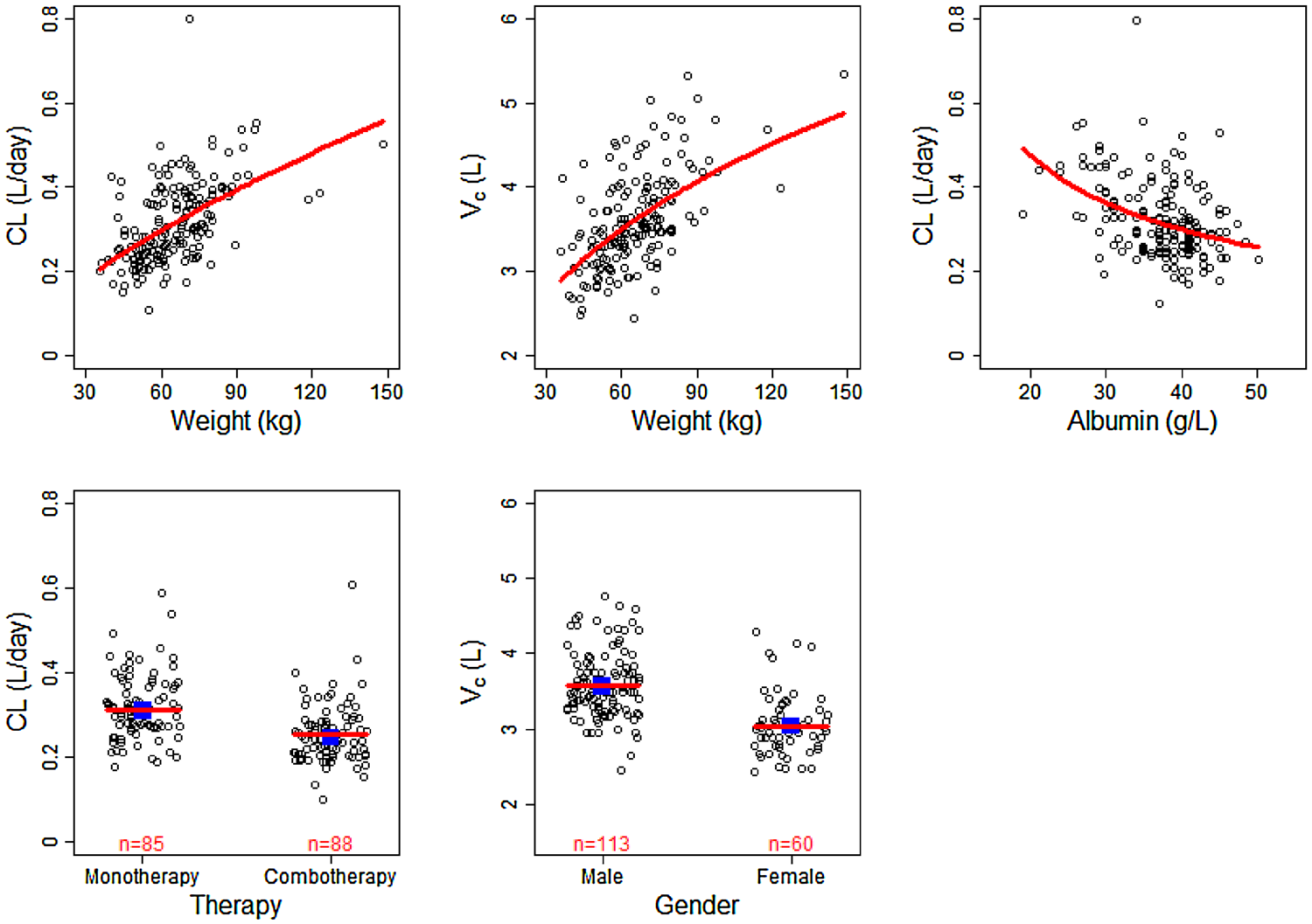
Pharmacokinetic parameter–covariate relationships for the final population pharmacokinetic model. Circles are the empirical Bayes estimates of individual PK parameters after correcting for all other covariates except for the one plotted in the *x*-axis. Blue squares represent the geometric mean within the group for categorical covariates. Red lines represent the typical (population) predicted parameter–covariate relationship based on the model. *CL* linear clearance, *V*_*c*_ central compartment volume, *PK* pharmacokinetics

General goodness-of-fit plots showed good agreement between predicted and observed concentrations of bemarituzumab (Supplemental Fig. 1a**)**, with no apparent bias in the residual plots (Supplemental Fig. 1b). The distribution of IIV is centered at zero and is normally distributed for all parameters (Supplemental Fig. 2). Pairwise correlations between the ETAs showed a slight correlation between CL and *V*_c_, which is consistent with the Ω matrix structure of the model (Supplemental Fig. 3). These results confirm that the structural model as well as the IIV and residual error models described the observed data well. The parameter–covariate relationships in the final popPK model, namely the effect of body weight on CL and *V*_c_, albumin and combotherapy/study on CL, and gender on *V*_c_ are illustrated in Fig. 1. Collectively, these plots indicate that the covariate model accurately described the relationships between individual PK parameters and covariates in the final popPK model. The pcVPC plots (Supplemental Fig. 4) and NPC (data not shown) showed that the final popPK model could adequately reproduce the central tendency and variability of the bemarituzumab serum concentrations across all studies.

### Sensitivity analysis

The sensitivity analysis showed that body weight was the most influential covariate on bemarituzumab exposure (Supplemental Fig. 5). Compared with a typical patient with a body weight of 64 kg, patients with body weight in the 10th percentile (45 kg) and 90th percentile (79 kg) of the GEA population were expected to have 13.4–18.0% lower and 7.0–12.1% higher steady-state exposures (AUC_ss_, *C*_max,ss_, and *C*_trough,ss_). Baseline albumin had a modest effect on bemarituzumab exposure, patients with albumin at the 10th percentile (30 g/L) and 90th percentile (44 g/L) of the GEA population were expected to have 5.7–18.9% lower and 3.9–13.2% higher steady-state exposures. Patients administrated with combotherapy were expected to have 18.8% higher AUC_ss_, 8.6% higher *C*_max,ss_, and 28.6% higher *C*_trough,ss_ compared to patients administered bemarituzumab as monotherapy. Compared to males, females were expected to have 0.9% higher AUC_ss_, 11.3% higher *C*_max,ss_, and 3.8% lower *C*_trough,ss_.

Overall, the differences in exposure due to these significant covariates were within the overall variability of exposure in the GEA population, which was − 39.7% to + 66.0%, − 27.0% to + 45.4% and − 56.2% to + 92.4% for the 5th to 95th percentiles of the population relative to the typical values of AUC_ss_, *C*_max,ss_, and *C*_trough,ss_, respectively (Supplemental Fig. 5).

### Population PK model simulations

The simulated geometric mean (5th percentile, 95th percentile) steady-state exposures of the GEA population following the target dose were 2805 (1578, 4348) µg*day/mL, 401 (277, 553) µg/mL, and 125 (50, 218) µg/mL for AUC_ss_, *C*_max,ss_, and *C*_trough,ss_, respectively.

**Fig. 2:**
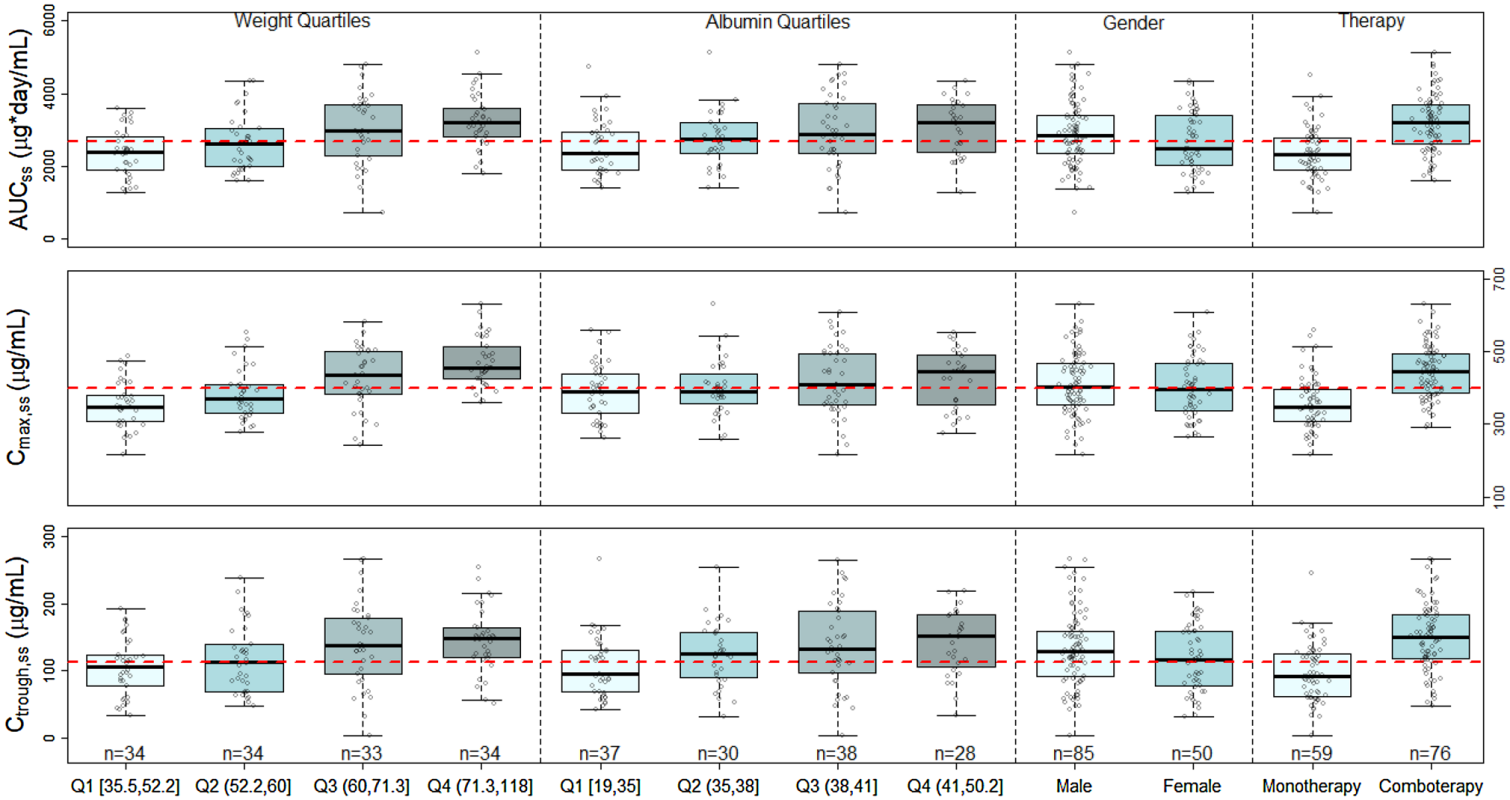
Simulated steady-state exposures of bemarituzumab stratified by significant covariates included in the final population pharmacokinetic model. Circles are the simulated steady-state bemarituzumab exposure in individual patients. The boxes represent the 25th–75th percentiles (the interquartile range). The solid black horizontal line in the middle of each box represents the median. The whiskers represent the range of data points within 1.5 times the interquartile range. The dashed red horizontal line represents the geometric mean of the GEA population. *AUC*_*ss*_ area under curve at steady-state, *C*_*max,ss*_ maximum concentration at steady-state, *C*_*trough,ss*_ trough concentration at steady-state, *n* number of patients, *Q* quartile, *GEA* gastric and gastroesophageal junction adenocarcinoma

The geometric mean simulated AUC_ss_, *C*_max,ss_ and *C*_trough,ss_ in the lowest (Q1) or highest (Q4) quartile were up to 15.0% lower and 20.9% higher for body weight and 13.7% lower and 16.8% higher for albumin, respectively, compared with those of the GEA population. The impact of gender on *V*_c_ resulted in 7.87% lower AUC_ss_, 2.03% lower *C*_max,ss_, and 8.68% lower *C*_trough,ss_ in females (*N* = 50) compared to with those in males (*N* = 85), while the impact of combotherapy (FIGHT, N=76) on CL resulted in 37.0% higher AUC_ss_, 25.1% higher *C*_max,ss_, and 64.3% higher *C*_trough,ss_ compared to monotherapy (FPA144-001 and FPA144-002, *N* = 59) (Fig. 2).

**Fig. 3:**
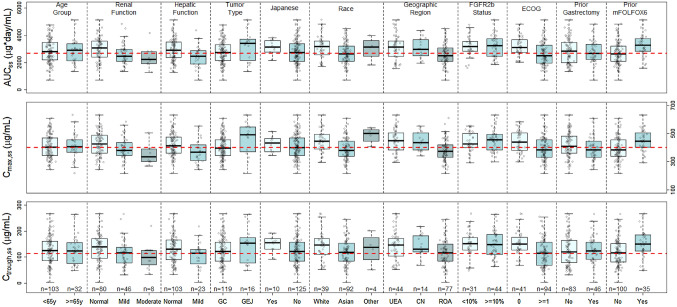
Simulated steady-state exposures of bemarituzumab stratified by non-significant covariates included in the population pharmacokinetic analysis. Circles are the simulated steady-state bemarituzumab exposure in individual patients. The boxes represent the 25th–75th percentiles (the interquartile range). The solid black horizontal line in the middle of each box represents the median. The whiskers represent the range of data points within 1.5 times the interquartile range. The dashed red horizontal line represents the geometric mean of the GEA population. *AUC*_*ss*_ area under curve at steady-state, *C*_*max,ss*_ maximum concentration at steady-state, *C*_*trough,ss*_ trough concentration at steady-state, *y* year, *n* number of patients, *UEA* US, Europe, and Australia, *CN* China mainland, *ROA* rest of Asia, *GC* gastric cancer, *GEJ* gastroesophageal junction adenocarcinoma, *GEA* gastric and gastroesophageal junction adenocarcinoma

The predicted steady-state exposure metrics were also analyzed for the covariate subgroups to evaluate the need for dose adjustment in patient subgroups of interest (Fig. 3). The geometric mean simulated AUC_ss_, *C*_max,ss_, and *C*_trough,ss_ in mild (*N* = 46) or moderate (*N* = 8) renal impairment were up to 14.6% lower or 23.8% lower, respectively, compared with those of patients with normal renal function (*N* = 80). Compared to patients with normal hepatic function (*N* = 103), patients with mild hepatic impairment (*N* = 23) were expected to have 17.9% lower AUC_ss_, 12.4% lower *C*_max,ss_, and 27.7% lower *C*_trough,ss_. The modest differences in the geometric mean AUC_ss_, *C*_max,ss_, and *C*_trough,ss_ were predicted across geographic regions (− 10.1% to 19.6%), between ECOG performance status (− 9.99% to + 27.3%), Japanese and non-Japanese (− 1.86% to + 26.4%), and with or without a single dose of mFOLFOX6 prior to randomization (− 7.44% to 24.7%), compared with the GEA population geometric mean (Fig. 3). The relatively small differences in geometric mean simulated AUC_ss_, *C*_max,ss_, and *C*_trough,ss_ were predicted between elderly patients ≥ 65 years and adults < 65 years (− 0.474% to + 1.54%), White and Asian (− 6.5% to + 16.7%), with or without prior gastrectomy (− 4.44% to + 2.55%), IHC FGFR2b ≥ 10% and FGFR2b 1–9% (− 1.41% to + 1.00%), and GC and GEJ (− 1.95% to + 15.8%), compared with the GEA population geometric mean (Fig. 3). These differences in exposures across covariate subgroups were small compared with the overall variability of exposures in the GEA population (− 27.0% to + 92.4%) (Supplemental Fig. 5).

## Discussion

The final popPK model described the bemarituzumab PK data well, as assessed by diagnostic goodness-of-fit plots, individual fits, pcVPC, NPC, shrinkage, and non-parametric bootstrap results. The sensitivity analysis and population simulations support the decision to test the same dose and regimen used in the phase 2 FIGHT trial for a future phase 3 trial in the GEA population with FGFR2b over expression in combination with mFOLFOX6 without any dose adjustment.

Bajaj et al. summarized the most common covariates among 23 mAbs approved for use in at least one oncology indication were baseline body weight and gender on CL and *V*_c_ as well as baseline albumin on CL [12]. In our current popPK model for bemarituzumab, body weight, baseline albumin, gender, and combotherapy/study were identified as statistically significant covariates which are consistent with what was previously reported, except for the addition of combotherapy/study. Body weight was identified as a significant covariate on CL and *V*_c_. Albumin and combotherapy/study were significant covariates on CL, and gender was a significant covariate on *V*_c_. In comparison with the popPK analysis reported previously [5], combotherapy/study was identified as a new significant covariate, as the previous popPK analysis did not include any samples from combination treatment or first-line patients with GEA [5]. Other covariates evaluated, including age, race, Japanese ethnicity, geographic region, eGFR, alkaline phosphatase, aspartate aminotransferase, alanine aminotransferase, total bilirubin levels, total protein, hepatic function based on NCI-ODWG, lactate dehydrogenase, ECOG performance status, tumor size, tumor type, FGFR2b status, prior gastrectomy, and administration of a single dose of mFOLFOX6 prior to randomization did not show a statistically significant impact on the PK of bemarituzumab. An immunogenicity impact assessment was not performed since no patients developed anti-bemarituzumab antibodies post-bemarituzumab treatment.

We previously compared the observed serum concentrations from combotherapy to simulated PK profiles from monotherapy and concluded that they are within a similar range [5]. However, considering the small patient population (12 patients), a covariate analysis between combotherapy and monotherapy was not performed in the previous analysis [5]. Seventy-six patients in the first-line setting were treated with bemarituzumab in combination with mFOLFOX6 in the phase 2 FIGHT trial, and this combotherapy/study was identified as a covariate for CL. This finding of combotherapy as a covariate was unexpected because bemarituzumab and mFOLFOX6 have distinct mechanisms of actions, metabolism, and clearance mechanisms. Bemarituzumab is cleared from the body via target-mediated clearance (specific clearance) and proteolytic catabolism (non-specific clearance). Therefore, most likely, the finding resulted from different patient populations between monotherapy (FPA144-001 and FPA144-002) and combotherapy (FIGHT) studies. Studies FPA144-001 and FPA144-002 enrolled later line patients with solid tumor including GEA, and phase 2 of FIGHT enrolled patients with previously untreated advanced first-line patients with FGFR2b overexpressing GEA only except advanced gastrointestinal (GI) tumors for phase 1 patients. New data from future trials will provide more insights for us to understand the observation.

Since bemarituzumab has nonlinear clearance due to target-mediated CL based on the phase 1 dose-escalation study FPA144-001 (0.3–15 mg/kg), an important question is whether the expression level of FGFR2b has any impact on the PK of bemarituzumab. We previously reported that FGFR2b status at baseline (high vs others) in patients with late line GEA using bemarituzumab as monotherapy in the FPA144-001 trial was not a covariate for PK. Results from the FPA144-001 and FIGHT trials could not be combined to evaluate whether FGFR2b status was a covariate for PK due to slight differences in the IHC assays for each trial. Therefore, the data from the FIGHT trial were used to perform the analysis alone and showed that the *V*_max_-FGFR2b expression relationship was not significant at *p* < 0.05 and, therefore, was not carried forward to the forward addition covariate search. This observation could be confounded by the fact that patients enrolled in the FIGHT trial were administered a dose in the linear dose range. Since the target dose was in the linear dose range, this observation supports the fact that expression levels of FGFR2b will not require any dose justification, at least for the target dose tested in the FIGHT trial.

The sensitivity analysis showed that, compared with a typical male patient on monotherapy with a body weight of 64 kg and albumin of 38 g/L, patients with body weight and baseline albumin at the 10th percentile and 90th percentile of the GEA population were expected to have < 20% lower or higher steady-state exposures (Supplementary Fig. 5). Patients with combotherapy were expected to have 8.6–28.6% higher steady-state exposures compared to patients with monotherapy, while the effect of gender on bemarituzumab exposure was relatively small (0.9–11.3%).

Population PK simulation based on the final model for the GEA population showed that moderate exposure differences between covariate subgroups examined were all within ± 30% except for combotherapy/study (Figs. 2, 3). Although the difference between monotherapy (FPA144-001 and FPA144-002) and combotherapy (FIGHT) is greater than 30%, this should not impact the dose and regimen selection for a future phase 3 trial, as it will be designed to repeat what was observed in the previous FIGHT trial and bemarituzumab will be used with mFOLFOX6. The primary analysis results (data cut-off date 23 September 2020) from phase 2 of the FIGHT study showed that bemarituzumab combined with mFOLFOX6 led to clinically meaningful improvements in PFS (HR = 0.68, 95% CI 0.44–1.04; *p* = 0.07) and OS (HR = 0.58, 95% CI 0.35–0.95; *p* = 0.03) compared with placebo plus mFOLFOX6 [4]. ORR also improved 13% in the bemarituzumab arm (53 vs 40%) [4]. The frequency of serious adverse events was similar in the study arms (31.6 vs 36.4%). However, adverse event-related discontinuation occurred more often in the bemarituzumab arm (36.4 vs 5.2%). The length of therapy was similar in both arms (24 vs 26 weeks). All patients with partial response (PR) achieved targeted *C*_trough_ of ≥ 60 μg/mL based on the first-observed *C*_trough_ on day 28 (Cycle 3 Day 1) [5]. In conclusion, the FIGHT trial had clinically meaningful and statistically significant improvements in PFS, OS, and ORR with manageable safety, which supported the selection of the current target dose and regimen in combination with mFOLFOX6 in the same population.

Given the low IIV of bemarituzumab key PK parameters (CL and *V*_c_) and the small or moderate effect of all statistically significant covariates on bemarituzumab exposure (AUC_ss_, *C*_max,ss_, and *C*_trough,ss_), no covariate is expected to have clinically meaningful effects on bemarituzumab exposure (Table 2). Additionally, the differences in exposures across covariate subgroups were small compared with the overall variability of exposures in the GEA population (Supplementary Fig. 5). Therefore, no dose adjustment is warranted for a future phase 3 trial in combination with mFOLFOX6 for a patient population similar to the FIGHT trial. However, further assessment is needed once additional data are available representing larger sample sizes.

## Supplementary Information

Below is the link to the electronic supplementary material.

Supplementary file1 (DOCX 227 KB)

## Data Availability

The datasets generated during and/or analyzed during the current study are available from the corresponding author on reasonable request.

## Code availability

Model parameter estimation and model evaluation were implemented with NONMEM 7, Version 7.4.3 (ICON Development Solutions. Ellicott City, MD, USA) [9] with GNU Fortran 95 Compiler (Version 4.6), Perl-Speaks-NONMEM (PsN) Version 4.2 (Uppsala University, Sweden) [10, 11], and R (version 3.5.3).
