# Supplementary material for: Covariate effects and population pharmacokinetic analysis of the anti-FGFR2b antibody bemarituzumab in patients from phase 1 to phase 2 trials
Source: Cancer Chemother Pharmacol. 2021 Aug 12;88(5):899–910. doi: 10.1007/s00280-021-04333-y (PMC8484135; doi:10.1007/s00280-021-04333-y)

# SUPPLEMENTARY MATERIAL

Table 1 Summary of studies included in the population PK analysis

| **Trial No.** | **Dose Regimen** | **N** | **Trial Description** | **PK Sampling Design** | **ADA Sampling Design** |
| --- | --- | --- | --- | --- | --- |
| FPA144-001 (NCT02318329) | Every two weeks in 28-day cycles at multiple dose levels (0.3 mg/kg, 1 mg/kg, 3 mg/kg, 6 mg/kg, 10 mg/kg, and 15 mg/kg) | 79 | A Phase 1 Open-Label, Dose-Finding Study Evaluating Safety and Pharmacokinetics of FPA144 in Patients with Advanced Solid Tumors | Parts 1A and 1B: Rich sampling with ≤9 samples for Cycle 1 and sparse sampling for other cycles. Part 2: rich sampling with ≤5 samples for Cycle 1 and sparse sampling for other cycles. | Anti-drug antibody samples were collected prior to dosing on Day 1 of Cycles 1 to 5, every other cycle after Cycle 5, and at the EOT. |
| FPA144-002 | Every two weeks in 28-day cycles at 2 dose levels (6 mg/kg and 15 mg/kg) | 6 | A Phase 1 Open-Label, Dose-Finding Study Evaluating Safety and Pharmacokinetics of FPA144 in Japanese Patients with Advanced Gastric or Gastroesophageal Cancer | Rich samplings with ≤14 samples for Cycle 1 and sparse sampling for patients that are continuing in the Extended Treatment Period. | Anti-drug antibody samples were collected prior to dosing on Day 1 and Day 15 on Cycle 1 and prior dose of each cycle and at the EOT. |
| FPA144-004 (NCT03343301) | 15 mg/kg Q2W with 1 additional dose of 7.5 mg/kg on Cycle 1 Day 8 in 14-day cycles | 12 | A multi-center study to evaluate the safety, tolerability, PK, and PD of FPA144 in combination with 5‑fluorouracil, leucovorin and oxaliplatin (mFOLFOX6) for patients with gastrointestinal malignancies. | Rich sampling with <9 samples for Cycles 1 and 2 and sparse sampling for other cycles. | Anti-drug antibody samples were collected prior to dosing on Cycle 1, 2, 3, 7, 10 and at the EOT. |
| FPA144-004 (NCT03694522) | 15 mg/kg Q2W with 1 additional dose of 7.5 mg/kg on Cycle 1 Day 8 in 14-day cycles | 76 | A global, randomized, double-blind, controlled study to evaluate the efficacy of bemarituzumab + mFOLFOX6 versus placebo + mFOLFOX6 in patients with FGFR2 selected Gastric Cancer (as determined by prospective IHC FGFR2b overexpression and/or a ctDNA blood assay demonstrating FGFR2 gene amplification) | Sparse sampling with total of 7 collection time points for a patient stay on the study ≥17 Cycles. | Anti-drug antibody samples were collected prior to dosing on Cycle 1, 3, 5, 9, 17 and at the EOT. |

Abbreviations: Q2W, once every two weeks; C, cycle; D, day.

**Fig. 1 Diagnostic plots for the final population pharmacokinetic model**

Observed versus individual predicted concentrations (top left) and observed versus population predicted (PRED) concentrations (top right) for the final popPK model. Points are individual data, red lines represent the line of unity, and blue dashed lines are lowess smooth curves showing the relationship between the two variables. Conditional weighted residuals (CWRES) versus time (bottom left) and population predicted (PRED; bottom right). Points are individual data. Red solid lines represent the horizontal line at zero. Green dotted lines represent |CWRES| of 5. The blue dashed lines are lowess smooth curves showing the relationship between the two variables.

**Fig. 2 ETA histograms for the final population pharmacokinetic model**

Red solid lines represent the unit line at zero. Blue dashed lines represent the median value of ETAs.

Abbreviations: etaV_M_, individual ETA for V_M_; etaCL, individual ETA for CL; etaV_c_, individual ETA for V_c_; etaVp, individual ETA for Vp; ETA, random effects; CL, linear clearance; V_c_, central compartment volume; V_p_, peripheral compartment volume; V_M_, maximum drug elimination by nonlinear clearance.

**Fig. 3 Pairwise correlation plots of the individual ETA estimates from the final population pharmacokinetic model**

Points are the post-hoc estimates from NONMEM. Red lines are smooth curves (lowess) showing the relationship between two variables.

Abbreviations: etaCL, individual ETA for CL; etaV_c_, individual ETA for V_c_; etaV_p_, individual ETA for V_p_; etaV_M_, individual ETA for V_M_; ETA, random effects; CL, linear clearance; V_c_, central compartment volume; V_p_, peripheral compartment volume; V_M_, maximum drug elimination by nonlinear clearance.

**Fig. 4 pcVPC of bemarituzumab serum concentration-time profiles across all studies**

Black open circle are individual observed concentrations, solid red lines represent the median observed concentrations, and dashed red lines represent 2.5%ile and 97.5%iles of the observed concentrations over time. Pink shaded areas represent the 95% CI of the predicted median concentrations, and blue shaded areas represent the 95% CI of the predicted 2.5^th^ and 97.5^th^ %iles of the concentrations over time. The top panel included all patients (n=173) while the bottom panel included patients with GEA (N=135). 11 data points after 56 days were not shown.

Abbreviations: pcVPC, prediction-corrected visual predictive check; GEA, gastric and gastroesophageal junction adenocarcinoma.

Fig. 5 Sensitivity analysis plot comparing the effect of significant covariates on bemarituzumab steady-state exposure (AUC_ss_, C_max,ss_, and C_trough,ss_)

The black vertical line refers to the predicted exposure (AUC_ss_, C_max,ss_, and C_trough,ss_) of bemarituzumab in a typical patient after 15 mg/kg Q2W with 1 additional dose of bemarituzumab 7.5 mg/kg on Cycle 1 Day 8 for 1 year which serve as the reference values. All percentage values shown in each plot are the relative changes in exposure relative to the reference value. The black shaded bar with values at each end shows the 5th to 95th percentile exposure range across the GEA patient population. Each blue shaded bar represents the magnitude of influence of the respective covariate on the exposure. The length of each bar represents the range of predicted bemarituzumab exposure between the high/low or possible values of the covariate (indicated at each end of the bar). The covariates shown in each plot are ordered from the most influential covariate at the top to the least influential covariate at the bottom.

Abbreviations: AUC_ss_, area under curve at steady-state; C_max,ss_, peak concentration at steady-state; C_trough,ss_, trough concentration at steady-state; PI, prediction interval; Q2W, once every two weeks; GEA, gastric and gastroesophageal junction adenocarcinoma.

Fig. 1 Diagnostic plots for the final population pharmacokinetic model


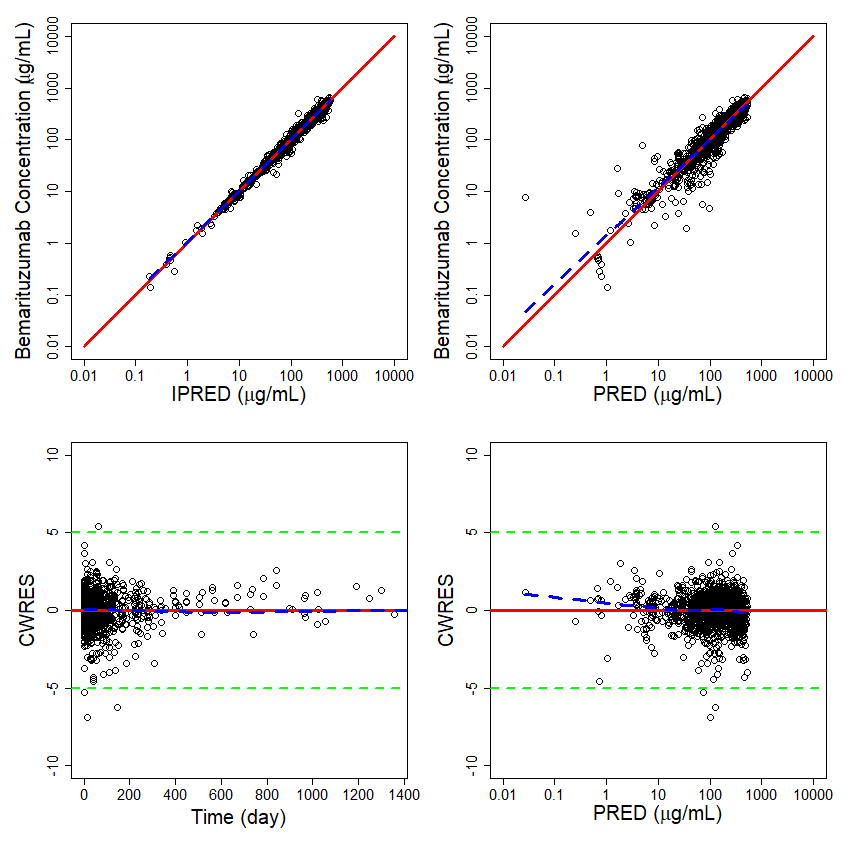


Fig. 2 ETA histograms for the final population pharmacokinetic model


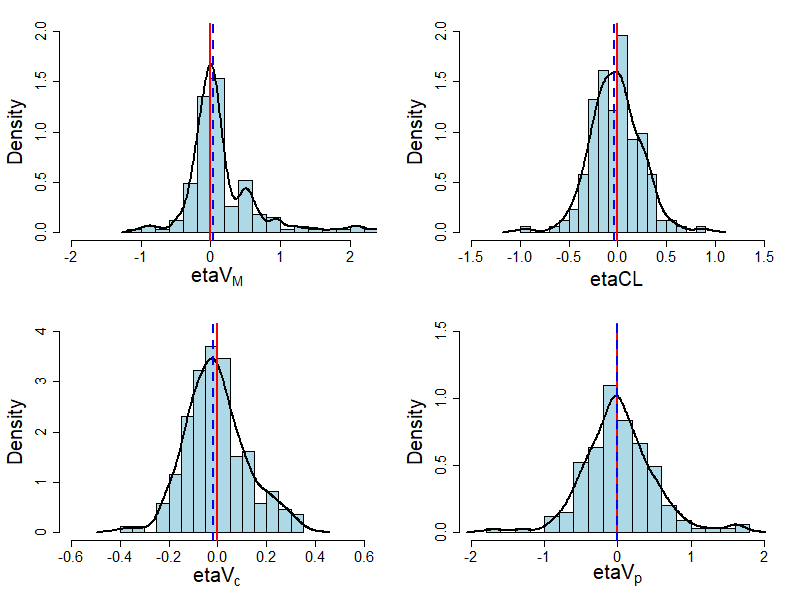


Fig. 3 Pairwise correlation plots of the individual ETA estimates from the final population pharmacokinetic model


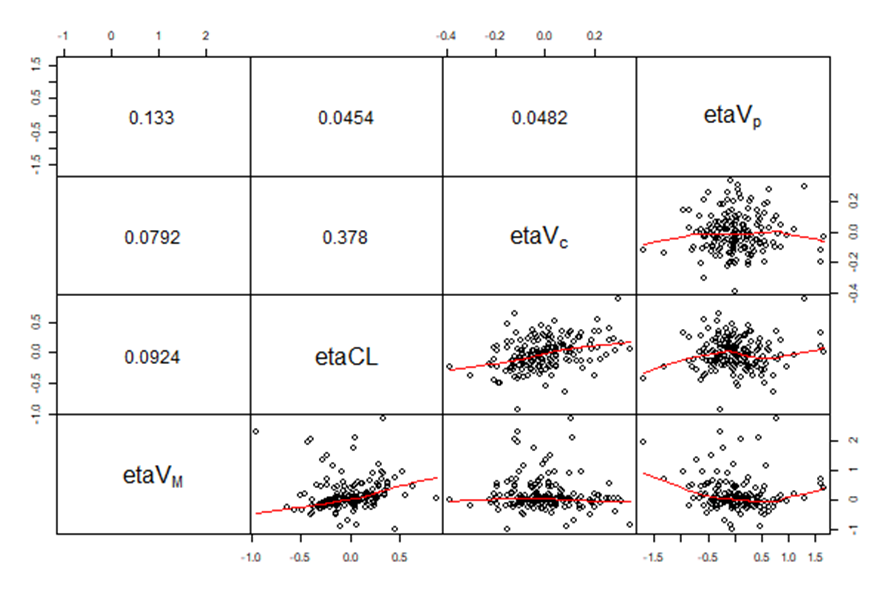


Fig. 4 pcVPC of bemarituzumab serum concentration-time profiles across all studies


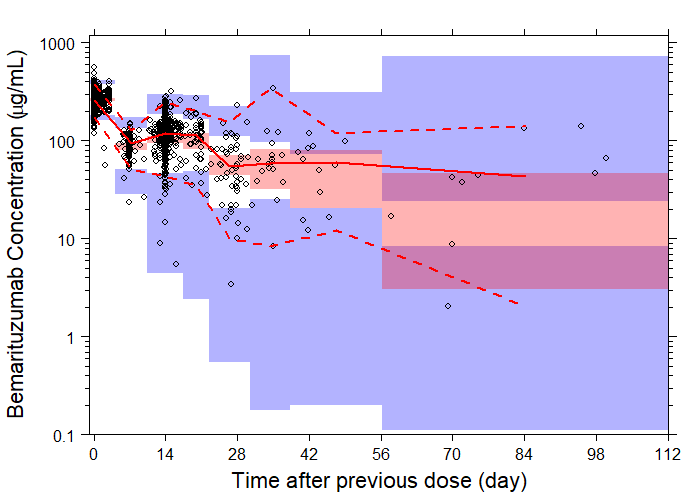


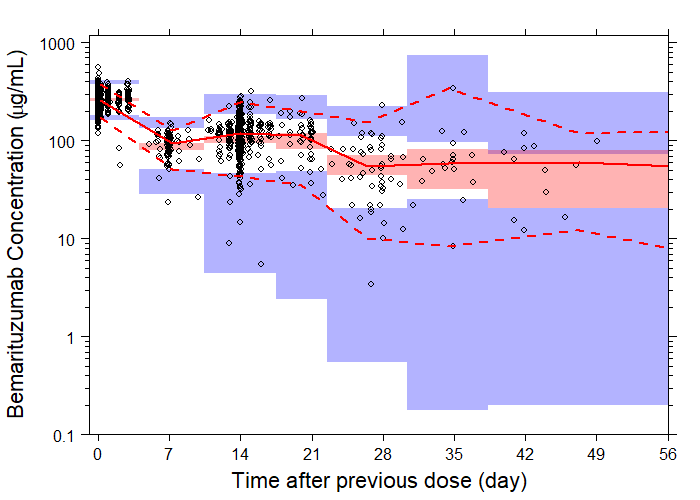


Fig. 5 Sensitivity analysis plot comparing the effect of significant covariates on bemarituzumab steady-state exposure (AUC_ss_, C_max,ss_, and C_trough,ss_)


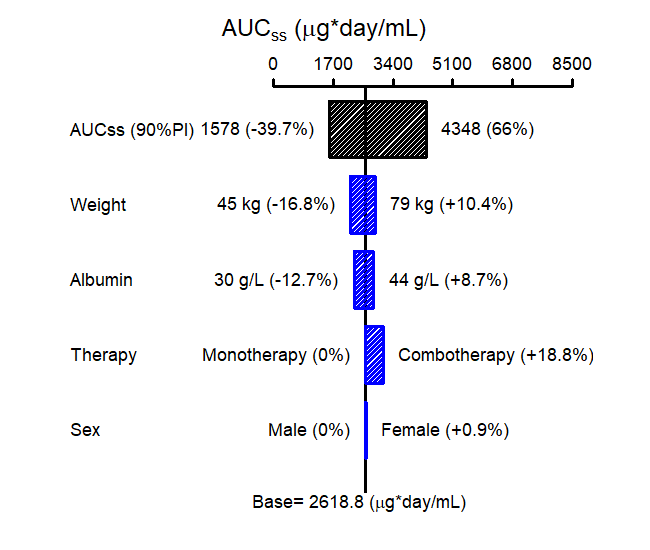

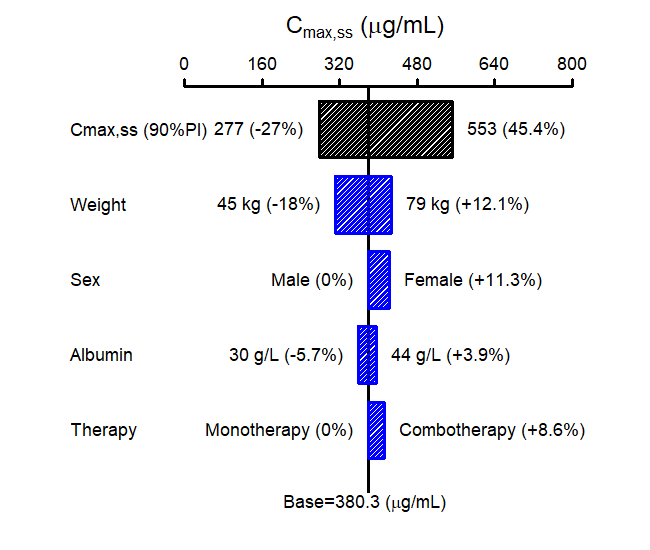


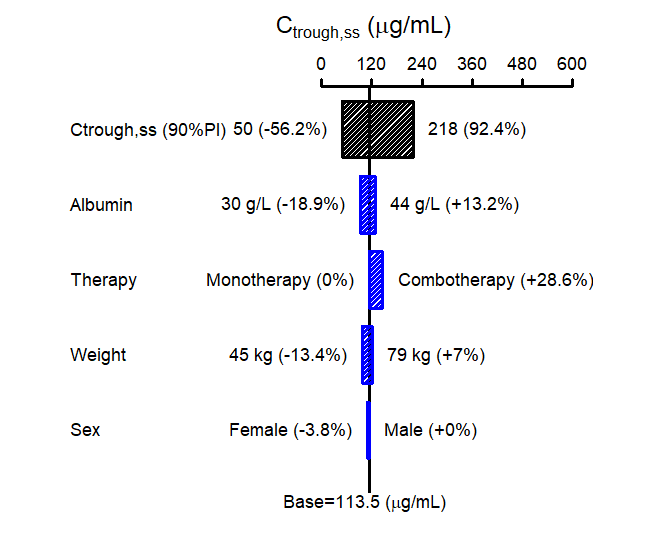

Supplement: Supplementary file 1 — Supplementary file1 (DOCX 227 KB) [file 280_2021_4333_MOESM1_ESM.docx]
